# Maternal distress and parenting during COVID-19: differential effects related to pre-pandemic distress?

**DOI:** 10.1186/s12888-023-04867-w

**Published:** 2023-05-29

**Authors:** Ann Low, Yue Yu, Lit Wee Sim, Jean Francois Bureau, Ngiap Chuan Tan, Helen Chen, Yang Yang, Bobby Cheon, Kerry Lee, Marian Bakermans-Kranenburg, Stella Tsotsi, Anne Rifkin-Graboi

**Affiliations:** 1 Centre for Research in Child Development, National Institute of Education, Nanyang Technological University, Block 5, Level B3, Singapore, 637616 Singapore; 2 School of Psychology, Faculty of Social Sciences, University of Ottawa, 136 Jean-Jacques Lussier, Vanier Hall, Room 6005, Ottawa, ON K1N 6N5 Canada; 3 Present Address: Duke-National University of Singapore, 8 College Road, Singapore, 169857 Singapore; 4 SingHealth Polyclinics, Connection One, 167 Jalan Bukit Merah, Tower 5, #15-10, Singapore, 150167 Singapore; 5 Department of Psychological Medicine, KK Women and Children’s Hospital, 100 Bukit Timah, 17 Road, Singapore, 229899 Singapore; 6 Eunice Kennedy Shriver National Institute of Child Health and Human Development, National Institutes of Health, Bethesda, MD USA; 7 Department of Early Childhood Education, Education University of Hong Kong, New Territories, 10 Lo Ping Road, Tai Po, Hong Kong, Hong Kong SAR, China; 8 ISPA - University Institute of Psychological, Social and Life Sciences, 1149-041, Rua Jardim do Tabaco, 34, Lisbon, Portugal; 9 PROMENTA Research Centre, Department of Psychology, University of Oslo, Postboks 1094 Blindern, 0317 Oslo, Norway

**Keywords:** Risk factors, Mental health, Maternal sensitivity, COVID-19, Stress, Adversity, Pre-existing conditions

## Abstract

**Background:**

Distinguishing whether and how pre-existing characteristics impact maternal responses to adversity is difficult: Does prior well-being decrease the likelihood of encountering stressful experiences? Does it protect against adversity’s negative effects? We examine whether the interaction between relatively uniformly experienced adversity (due to COVID-19 experience) and individual variation in pre-existing (i.e., pre-pandemic onset) distress predicted mothers’ pandemic levels of distress and insensitive caregiving within a country reporting low COVID-19 death rates, and strict nationwide regulations.

**Method:**

Fifty-one Singaporean mothers and their preschool-aged children provided data across two waves. Pre- pandemic onset maternal distress (i.e., psychological distress, anxiety, and parenting stress) was captured via self-reports and maternal sensitivity was coded from videos. Measures were repeated after the pandemic’s onset along with questionnaires concerning perceived COVID-19 adversity (e.g., COVID-19’s impact upon stress caring for children, housework, job demands, etc.) and pandemic-related objective experiences (e.g., income, COVID-19 diagnoses, etc.). Regression analyses (SPSS v28) considered pre-pandemic onset maternal distress, COVID-19 stress, and their interaction upon post-pandemic onset maternal distress. Models were re-run with appropriate covariates (e.g., objective experience) when significant findings were observed. To rule out alternative models, follow up analyses (PROCESS Model) considered whether COVID-19 stress mediated pre- and post-pandemic onset associations. Models involving maternal sensitivity followed a similar data analytic plan.

**Results:**

Pre-pandemic maternal distress moderated the association between COVID-19 perceived stress and pandemic levels of maternal distress (β = 0.22*, p* < 0.01) but not pandemic assessed maternal sensitivity. Perceived COVID-19 stress significantly contributed to post-pandemic onset maternal distress for mothers with pre-pandemic onset distress scores above (β = 0.30, *p* = 0.05), but not below (β = 0.25, *p* = 0.24), the median. Objective COVID-19 adversity did not account for findings. Post-hoc analyses did not suggest mediation via COVID-19 stress from pre-pandemic to pandemic maternal distress.

**Conclusions:**

Pre-existing risk may interact with subsequent perceptions of adversity to impact well-being. In combination with existing research, this small study suggests prevention programs should focus upon managing concurrent mental health and may highlight the importance of enhanced screening and proactive coping programs for people entering high stress fields and/or phases of life.

**Supplementary Information:**

The online version contains supplementary material available at 10.1186/s12888-023-04867-w.

Everyone experiences difficult events, but pre-existing factors may influence how individuals react during such times. Indeed, some individuals are more susceptible to adversity than others [1]. For example, in the context of mental health, much research has examined the risk factors for posttraumatic stress disorder (PTSD), and a recent umbrella review has identified medical history and family psychiatric history as pre-existing factors that increase the likelihood of developing PTSD following a traumatic event [2]. However, a major limitation in research investigating pre-adversity risk factors is that it is difficult to identify whether these risk factors increased the likelihood of *exposure* to potentially adverse events, or if they increased *vulnerability* towards developing distress symptoms following exposure to the events [3].

Here we capitalize on the fact that within the Republic of Singapore, the emergence of the COVID-19 pandemic and corresponding strictly enforced nationwide governmental restrictions in early 2020 allowed for an approximation of a “natural experiment” examining the interaction between pre-existing emotional states and stress exposure amongst middle to upper class parents of young children. That is, all such parents received relatively similar *exposure* to the pandemic and associated restrictions regardless of pre-existing mental health and parenting factors, enabling us to study the differential impact of similar exposure on maternal distress and parenting.

First, throughout the time investigated in this study (February 2019 to January 2021, see Fig. 1), compared to other countries, relatively few people had direct exposure to COVID-19 severe illness (see Additional File 1) [4]. This enables us to focus on the *mental* health impact from COVID-related fear and restrictions, without the potential confound of *physical* health impacts from being infected and falling ill.

Second, unlike in some countries, where rules varied by state, province, county, or even city, in Singapore regulations occurred across the entire population of roughly 5.686 million people, were highly enforced, and travel outside of this small country (i.e., maximum width = 50 km and maximum length = 26 km) was substantially curtailed. Thus, comparatively speaking, all Singaporeans experienced relatively similar COVID-19 regulations (see Additional File 1 for greater detail), though the effects were likely felt most severely by those at the lowest income brackets [5]. In addition, these restrictions were clearly demarcated by different phases reinforced by governmental campaigns and signage.

Expectedly, in one retrospective study Singaporean parents reported experiencing the greatest psychological distress during a phase of the pandemic with the most substantial restrictions [6]. Likewise, worldwide, there is evidence that the COVID-19 pandemic had a significant impact on people’s mental health [7, 8]. Depressive and anxiety symptoms were especially high among parents and pregnant women, which is related to lack of healthcare services during pregnancy, home-schooling, and loss of social support [9].

Whether or not changes in pre- to post-pandemic onset mental health are influenced by prior mental states and experience is an important question for many reasons including that it may inform our understanding of the association between adversity, depression, anxiety, and insensitive parenting. On the one hand, work across three Canadian cohort studies suggests that pre-existing adversity, in the form of retrospectively reported comparatively low socioeconomic status and pre-pandemic childhood mental health diagnoses, served as risk factors for material deprivation during COVID-19, which in turn predicted parental well-being during a later phase of the pandemic [10].

However, another study examining the change in depression amongst roughly 200 British mothers of school children found that neither higher levels of depression assessed prenatally, well before COVID-19’s onset, nor the quantity of COVID-19 stressful experiences moderated change in depressive symptomatology, although those who lived in less deprived postal codes, perhaps surprisingly, appeared to show more of an increase in depressive symptoms; following a lock-down their symptomatology appeared similar to those living in more deprived areas [11]. Similarly, Robinson et al.’s review and meta-analysis [8] reports no changes in mental health problems pre-and post- pandemic onset amongst samples with pre-existing mental health conditions but increases in mental health symptoms in populations without such pre-existing conditions.

Still, within Singapore there is some evidence that higher levels of pre-pandemic adversity may lead to the experience of greater distress. A recently published Singaporean study investigated how experiences related to COVID-19 influenced changes in levels of depression and anxiety among low-income mothers of primary school children before and after the height of the pandemic restrictions in Singapore. While job impermanence was directly associated with greater depressive symptoms, the effects of other COVID-19 related stressors (e.g., job loss) were moderated by marital status and/or mothers’ levels of hope. Specifically, the loss of an income earner in the household led to greater depression only for single mothers, and to greater anxiety only for single mothers or mothers with low hope scores. Similarly, mothers’ job loss led to greater depression only for mothers with low hope scores [12]. These findings suggest that factors such as marital support or internal resilience may buffer against stressful experiences brought on by COVID-19. However, the study did not examine the potential moderation by pre-pandemic maternal mental health, nor did it consider observed parenting.

In addition, none of the aforementioned studies directly examined the interaction between pre-pandemic distress and perceptions of COVID-19 related stress, per se, upon the association between pre- and post-pandemic distress within a community sample. That is, while they examined pre- and post- pandemic levels of change as a function of pre-existing risk, they did not assess whether pre-existing psychological health interacted with COVID-19 perceived experience to predict well-being or distress during the pandemic.

Still, there is reason to think that perceptions about the pandemic may predict emotional distress. One study from the Netherlands examined lockdown-related changes in parental negative feelings (anxiety, depression, hostility and interpersonal sensitivity) and perceived stress among a group of parents of 10–13-year-old children. They found that perceived stress mediated the relation between pre-pandemic negative feelings and lockdown-related increase in negative feelings: Higher negative feelings prior to the lockdown were related to more perceived stress during the lockdown, which in turn was associated with an increase in parental negative feelings [13]. Similarly, another study from Germany showed that appraisals of the pandemic predicted mental health and life experiences during the pandemic: People who reported more negative appraisals at the start of the pandemic reported more negative affect and stressful events later on [14].

These seemingly contradictory findings from different studies may result from heterogeneity in COVID-related experiences and restrictive measures among different locations and across time. As mentioned in the meta-analysis from Robinson and colleagues, there is large heterogeneity in the mental health levels reported across studies, and the levels also change alongside the development of the pandemic [8]. Also, participants for most COVID-related mental health research came from either Europe and North America, or from China, and their experiences might not be generalizable to people living in other parts of the world. In this regard, the case of Singapore may provide unique insights as it is situated in the understudied area of South-East Asia, and experienced a well-defined lockdown period with uniformly enforced restrictive measures.

Another research gap we aim to address is the population of parents with young children. This is a group that is particularly vulnerable to COVID-related mental health problems [9], yet little is known about the underlying mechanisms. Indeed, a recent PubMed search (April 6^th^, 2022) with the jointly entered terms “parent,” “COVID” and “cohort” suggests that pre-post pandemic work has not specifically focused upon longitudinally assessing mothers of typically developing preschool aged children, despite the unique pressures such women may have faced balancing caregiving, work, and other responsibilities, and the well-known links between maternal mental health, parenting, and child outcomes [15, 16].

Aspects of parenting, such as maternal sensitivity, are also likely to be susceptible to the effects of stress. Higher levels of maternal sensitivity have been associated with different factors, including lower socioeconomic stress [17], lower parenting stress and better mental health [18], as well as better executive function [19]. Maternal sensitivity also has long-lasting effects on offspring outcomes, such as children’s social and academic competence [20, 21]. Specific to the COVID-19 period, research has shown that COVID-related changes in children’s mental health are associated with parental autonomy support, parental need fulfilment, and parent–child conflict [22, 23]. Parenting practices themselves, such as autonomy support and behavioural control, also changed during lockdown [24]. Again, these studies focus on parents of school-aged children and adolescents, and it is still unclear whether they also apply to parents of preschool-aged children. Nevertheless, such work suggests that stress related to COVID-19 could potentially negatively and directly influence maternal sensitivity, perhaps especially for those already at risk, and given associations between sensitivity and offspring outcomes [20, 21], may also negatively impact child development.

The current study aims to investigate the effects of COVID-19 perceived stress on maternal distress (e.g., anxiety, comparatively high levels of general life-stress and/or parenting stress) and caregiving sensitivity in a middle to high socioeconomic status Singaporean sample, and to determine whether individual variation in maternal distress prior to the pandemic’s onset differentially influences the extent to which COVID-19 relates to post-pandemic onset levels of distress and sensitive care. The study takes advantage of data from a Two-Wave study, Singapore Parenting And Cognition in Early childhood (SPACE), in which parental distress was measured through self-report and maternal sensitivity was measured through observation before the COVID-19 pandemic in Wave One. The same parent–child dyads were engaged again during the pandemic in Wave Two, when they completed the same measures as well as a COVID-19 Questionnaire about perceptions and experiences of the pandemic and lockdown. Because parent–child dyads were recruited and first tested before the spread of COVID-19 in Singapore, and lockdown measures were enforced uniformly across the island nation, this study allows a unique opportunity to study the role of pre-existing mental health and parenting factors without them influencing the likelihood of adversity exposure. In keeping with past Singaporean research, COVID-19 stress is hypothesized to directly influence maternal distress [6], and for similar reasons maternal sensitivity. In addition, despite aforementioned research indicating less capacity for change in those at greatest risk [8], in keeping with a diathesis-stress model, effects were expected to be stronger for mothers with higher pre-pandemic distress.

## Method

### Participants and procedure

**Fig. 1:**
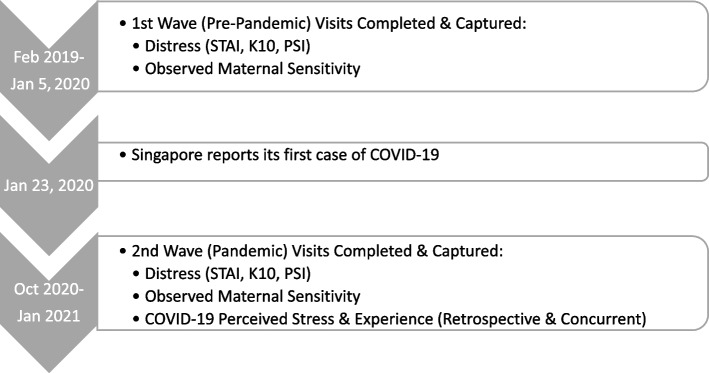
Study Design

Participants were mothers and their children taking part in the Two-Wave SPACE study (see Fig. 1 for the study overview). All mothers gave informed consent and children gave informed assent. This study was approved by the Nanyang Technological University’s Institutional Review Board (NTU-IRB Ref no. 2018–04-015), and all methods were performed in accordance with the relevant guidelines and regulations.

Criteria for participation were mother–child pairs with children aged 4–5 years with no known neurological or psychiatric disorders and no known head injuries, born from singleton pregnancies. Initially, interested dyads were recruited through 145 schools and organizations as well as outreach talks, with the aim of recruiting right-handed Singapore citizen or permanent resident children with exposure to both English and a Chinese language. However, as recruitment numbers were initially low, these language criteria were dropped and a social media campaign was also carried out to increase enrolment. Because the SPACE study was focused on normative child development, recruitment criteria focused on children with no requirements concerning psychiatric or neurological conditions in the mothers. Still, an examination of the Kessler Psychological Distress Scale (see below) suggested that within the current study, two mothers were likely to have severe or moderate mental illness at Wave One (but not Wave Two), and another two mothers were likely to have moderate mental illness at Wave Two (but not Wave One).

Sixty-seven dyads took part in the first visit, when the children (35 girls and 32 boys) were 4.5 years old on average (*SD* = 0.3). All except one initial visit were conducted in participants’ homes, and all visits were completed by 5^th^ January 2020, before Singapore initiated its disease outbreak response plan [25] and before the World Health Organization declared COVID-19 a pandemic [26]. At the time of the first visit, mothers were between 23 and 46 years old (*M* = 35.5, *SD* = 4.3). Singaporean residents are primarily ethnic Chinese (74.3%), followed by Malay (13.5%), and then Indian (9.0%); likewise the ethnicities represented by mothers in this study were primarily Chinese (88%) followed by Malay (7%) and then Indian (3%) and Javanese (1%), though the study population was more highly skewed towards ethnic Chinese inclusion, perhaps because of the initial language requirements.

All mothers had at least secondary school level qualifications, with 76.1% holding a university or postgraduate degree. High levels of maternal education are not unusual in Singapore; using data retrieved from the Singapore Department of Statistics [27], in 2021 the percentage of women aged 25–44 with University degrees or higher was roughly 59.6%. Still, this level of education was not specifically selected during recruitment.

The second visit took place approximately one year later when the children were 5.7 years old on average (*SD* = 0.4). Fifty-one dyads (76%) returned for this visit. All Wave Two visits were conducted at the lab, and took place from 30^th^ September 2020, which was several months after Singapore’s Circuit Breaker (additional information about COVID-19 phases in Singapore can be found in Additional File 2). All but one of the Wave Two visits were completed by December 2020 (98% were completed by December 14^th^ 2020), with one visit occurring in early January. As the COVID-19 vaccine only become available in Singapore on December 21^st^ 2020 and initial doses were reserved for front line or essential workers, it is unlikely that any participants had received the vaccine at the time of the Wave Two visit.

Participants completed all measures at both visits, except for the COVID-19 Questionnaire which was only completed at the second visit. All self-reported measures were completed using an online questionnaire. Comparisons between participants who completed both visits and those who completed only the first visit revealed no differences in maternal education, maternal year of birth, child age, child gender, as well as baseline maternal distress and observed maternal sensitivity (see Additional File 3).

## Study design

### Measures

#### Demographics

Child demographics (e.g., gender and age) and maternal demographics (e.g., age and education) were collected during the initial visit. To assess education level, participants were asked to report the highest level of education they had completed on a 5-point scale, from *Primary Level qualifications or below* (1) to *Postgraduate Level (Masters, PhD, or equivalent higher degree)* (5).

#### COVID-19 Questionnaire (COVID-19 stress)

As depicted in Fig. 1, COVID-19 Stress was assessed at one timepoint, namely during the Wave Two Visit, which occurred after the onset of COVID-19 in Singapore. For each item in the COVID-19 questionnaire, participants were asked to give separate ratings for three phases of the Pandemic: COVID-19’s Beginning, the Circuit Breaker (Lockdown), and Post-Circuit Breaker (see Additional File 2 for a description of the three phases). As such, participants gave retrospective accounts for the first two phases and a combination of retrospective and concurrent accounts for the last, Post-Circuit Breaker phase, which queried from “June 2020-current”. Questions were relevant to “perceived” and “objective” stress. The full questionnaire can be found in Additional File 4, and is a slightly modified version of a questionnaire developed by local researchers to investigate the effects of COVID-19 and lockdown measures in Singapore [6]. Participants indicated how much COVID-19 affected their stress levels on 18 items on a 10-point scale from *Not stressful at all* (0) to *Always stressful* (10)*,* or as *Not Applicable*. Examples of stressors include “caring for children,” “caring for the elderly,” “financial difficulties” and “limited opportunities to travel or go outside”. Responses of *Not Applicable* were given a score of zero, and items were averaged to produce an overall score as the COVID-19 stress scores were highly correlated across timepoints and with the average score (*r* = 0.73 to 0.95, *p* < 0.001; see Table 1). Internal reliability of the individual items within each time point, and in the composite with individual items from all three timepoints, was high (α ≥ 0.90). In addition, participants were asked to rate changes in objective experience, including loss of income, conflict with household members, COVID-19 media exposure, and COVID-19 diagnosis and/or isolation orders. None of the participants reported having been diagnosed with COVID-19.

**Table 1:** Bivariate Correlations Between COVID-19 Stress Scores for Each Time Period and with the Overall Average

	February to March	April to May	June onwards	Average
February to March	-	.81***	.73***	.92***
April to May		-	.83***	.95***
June onwards			-	.91***

^*t*^* p* < .10, * *p* < .05, ** *p* < .01, *** *p* < .001

#### Psychological distress

Psychological distress was measured using the Kessler Psychological Distress Scale (K10) [28], a 10-item measure about the frequency of which respondents experience distressing emotional states over the past 30 days. Participants rated items on a 5-point scale from *none of the time* (0) to *all of the time* (5). Items were summed to create a scale (α = 0.92 at Wave One and 0.91 at Wave Two), with higher scores indicating greater distress.

#### Parenting stress

Parenting stress was measured using the short form of the Parenting Stress Index – Fourth Edition (PSI) [29]. It consists of three subscales with 12 items each that are rated on a 5-point scale, from *strongly agree* (1) to *strongly disagree* (5). The Parental Distress (PD) subscale measures the level of distress that is directly related to parental obligations, the Parent–Child Dysfunctional Interaction (P-CDI) subscale focuses on negative aspects of the reciprocal interaction between parents and their children, and the Difficult Child (DC) subscale focuses on the child’s qualities that contribute to parenting difficulties. Items from each subscale were summed to produce total subscale scores for PD (α = 0.82 at Wave One and 0.85 at Wave Two), P-CDI (α = 0.85 at Wave One and 0.89 at Wave Two) and DC (α = 0.86 at Wave One and 0.90 at Wave Two), with higher scores indicating greater distress in each domain.

#### Anxiety

Anxiety proneness was measured using the Trait Anxiety subscale of the State-Trait Anxiety Inventory (STAI) [30]. Participants indicated how they generally felt by reporting how frequently they experienced anxious thoughts and feelings on 20 items using a 4-point scale from *Almost never* (1) to *Almost always* (5). Items were summed to produce a total score (α = 0.92 at Wave One and 0.90 at Wave Two), with higher scores indicating greater anxiety proneness.

#### Maternal sensitivity

Maternal sensitivity was assessed over four semi-structured scenarios of about three minutes each, which were designed to capture different types of natural occurrences. The “free-play” scenario involved mother–child dyads making their own puppets or picture frames or playing with existing ones. The “discipline” scenario involved cleaning up of the free-play materials, which required the mother to give instructions to her child. The “novelty” scenario involved a research assistant wearing a novel mask and offering a bag of fun masks to the dyad (in Wave One) or holding a walking and roaring toy dragon and offering a bag of toys to the dyad (Wave Two), which was potentially emotion-evoking with some free-play. The “teaching” scenario involved a math matching game that required the mother to teach her child the concept of addition. The four scenarios were counterbalanced, except with “discipline” always following “free-play.” Instructions were provided to mothers using a tablet with a video utilising closed caption instructions before each task, who would have to then convey the task to their child. The tablet was provided towards the end of the previous task, hence mothers had to divide their attention between the instructions and their child. Mothers were asked to behave as they normally would. The four scenarios were similar across timepoints for consistency.

Each scenario was coded separately using the preschool version of the shortened Maternal Behavioural Q-Set [31], which consisted of 25 items describing a range of maternal behaviours. Items were sorted into five equal piles based on how characteristic they were of the mother, from *Most unlike the mother* (1) to *Most like the mother* (5). Ratings were correlated with that of a prototypically sensitive mother and the resultant correlation coefficient scores which ranged from -1.0 to 1.0 indicated the maternal sensitivity scores, with higher scores indicating higher maternal sensitivity. Scores across the four tasks were averaged to produce a global maternal sensitivity score at the first (α = 0.73) and second (α = 0.74) timepoints. Data from Wave One was coded by two coders and data from Wave Two was coded by three main coders. Only one tape was coded by a fourth coder, as the participant was known to the three main coders. There was high interrater reliability [32] on the global sensitivity measure for at least 20% of the cases that were double coded, with intraclass correlation coefficients of 0.87 at both time points.

## Data analysis

First, preliminary analyses were conducted to reduce the maternal distress variables, thus reducing the risk of Type 1 error. The two mental health total scores (psychological distress, anxiety) and three parenting stress subscale scores (parental distress, parent–child dysfunctional interaction, child dysfunction) were standardized and submitted to a principal component analysis, separately for data from both timepoints, to determine whether a component maternal distress score could be used.

Second, first order relations among the composite maternal distress variable (hereafter, “Maternal Distress”), COVID-19 stress, and maternal sensitivity were conducted using Pearson correlations. This was followed by regression analyses to estimate the main and interactive effects of pre-pandemic maternal distress and COVID-19 stress on pandemic maternal distress and (separately) maternal sensitivity, with results reported in the main body of this paper. The sample size of the regression analyses depended on the number of cases with data in the outcome variable. For maternal distress, analyses were run on 49 cases, and on 50 cases for maternal sensitivity. For both analyses, predictor variables were standardized before entry into the first block of the analysis, and the interaction term was entered into the second block. For significant interactions, we investigated the pattern of the interaction effect by examining the regression coefficients of cases according to a median split, as well as for cases that were greater than one standard deviation above or below the median score of the moderator variable. Given past associations between maternal education and outcomes of interest, as well as consistency in maternal sensitivity levels across time points [33], models were re-run including maternal education, and/or pre-COVID 19 maternal sensitivity (see Additional File 5).

In addition, we took steps to further protect against the possibility that any observed interactions between pre-pandemic maternal distress and COVID-19 related stress could be explained by an association between differential experience during COVID-19. As noted above, in addition to asking participants about perceived COVID-19 stress, we also asked them about objective experience (i.e., loss of income, conflict with household members, COVID-19 media exposure, COVID-19 diagnosis and/or isolation orders). To ensure that these variables did not account for any of our findings, in cases where significant interactions between COVID-19 perceived stress and pre-pandemic maternal distress were uncovered in the regressions, models were re-run, including any objective experience that Pearson correlations found significantly related to COVID-19 related stress and/or maternal distress (see Additional File 6).We chose to re-run models with covariates rather than force all potential covariates into one model given the small sample size and concerns about Type 2 error.

Finally, after conducting the above analyses we examined our data in two additional ways to help rule out alternative hypotheses. First, to determine whether a mediational model would have better explained significant moderation, we assessed whether the observed relation between pre-pandemic maternal distress and pandemic maternal distress was mediated via COVID-19 stress. Second, to ensure that results in the maternal distress model were not unduly influenced by the high degree of association between the pre- and pandemic maternal distress variables, we repeated the initial model substituting the difference score between pre- and pandemic distress as our dependent variable. The high correlation between time points of our factor score was not surprising given test–retest reliability of the composite instruments (given high rates of test–retest reliability amongst the K10, STAI, and PSI (e.g. K10 *r* = 0.88-0.89 [34]; STAI *r* = 0.88 [35]; PSI *r* = 0.61-0.75 [36]).

Analyses were conducted with IBM SPSS Statistics Version 28 (SPSS Inc., Chicago, IL, USA). Mediation analyses were conducted using the PROCESS plugin by Andrew F. Hayes (http://www.processmacro.org/) with 5,000 bootstrap samples.

## Results

### Preliminary analyses

#### Reduction of maternal distress data

At both timepoints, the principal component analyses yielded a one-factor solution including all the entered variables (*Eigen* value > 1.0, representing 63.3% of the variance at the initial time point and 73.7% of the variance at the second time point; see Table 2 for the factor loadings). As such, the resulting component scores were used for subsequent analyses, with higher scores indicating greater maternal distress.

**Table 2:** Factor Loadings for One-Factor Solution for Maternal Distress

Items	Pre-pandemic maternal distress	Pandemic maternal distress
Psychological distress	.83	.83
Trait anxiety	.82	.83
Parental distress	.80	.87
Parent–child dysfunctional interaction	.78	.91
Child dysfunction	.76	.85
Total variance explained	63.3%	73.7%

### Main analyses

#### Correlations between maternal distress, maternal sensitivity, and COVID-19 stress

The Pearson correlations between the maternal distress variables, COVID-19 stress, and maternal sensitivity are presented in Table 3. Briefly, COVID-19 stress was marginally related to pre-pandemic maternal distress, and significantly to pandemic maternal distress. Maternal sensitivity across time points was significantly interrelated, as was maternal distress across timepoints. No other marginal or significant relations were observed.

**Table 3:** Pearson Correlations Between Wave One and Two Maternal Distress and Maternal Sensitivity Variables, and COVID-19 Stress

		Pre-pandemic		Pandemic		
		Maternal distress	Maternal sensitivity	Maternal distress	Maternal sensitivity	COVID-19 stress
Pre-pandemic	Maternal distress	-	.04	.84***	.07	.24^t^
	Maternal sensitivity		-	.04	.66***	.19
Pandemic	Maternal distress			-	.02	.38**
	Maternal sensitivity				-	.13

^*t*^* p* < .10, * *p* < .05, ** *p* < .01, *** *p* < .001

#### Relations between COVID-19 perceived and objective stress

As reported in Additional File 6, perceptions of COVID-19 stress was not significantly associated with knowing someone who had been diagnosed or received an isolation order, nor was it associated with COVID-19 media exposure. However, COVID-19 perceived stress was significantly associated with decreased household livelihood and increased household conflict (see Supplemental Table 4, Additional File 6).

#### Moderation analyses

##### COVID-19 Stress and Pre-Pandemic Maternal Distress in Relation to Pandemic Maternal Distress

Table 4 summarizes the results for the model examining pandemic maternal distress at Wave Two. The full model significantly explained 79.1% of the variance in maternal distress assessed during the pandemic (*p* < 0.001). There were significant main effects of COVID-19 stress (β = 0.23, *p* < 0.01) and pre-pandemic maternal distress (β = 0.76, *p* < 0.001). The interaction effect was also significant (β = 0.22, *p* < 0.01), and explained an additional 4.6% of variance (*p* < 0.01).

**Table 4:** Summary of Regression Analysis Predicting Pandemic Maternal Distress

Block	R^2^	ΔR^2^	*F* Change	β when first entered	β in final model
1. COVID-19 stress Pre-pandemic maternal distress	.746		67.47***	.20* .80***	.23** .76***
2. COVID-19 stress × Pre-pandemic maternal distress	.791	.046	9.87**	.22**	.22**

^*t*^* p* < .10, * *p* < .05, ** *p* < .01, *** *p* < .001

An examination of Cook’s Distances suggested that five cases may have had undue leverage, with two of these cases having scores that exceeded K10 clinical cut-offs at Wave One and one case that exceeded K10 clinical cut-offs at Wave Two. Hence, the above analysis was repeated without these three cases (see Supplementary Table 6, Additional File 7). Significant main effects remained, and the interaction effect was close to significance (i.e., p = 0.052).

**Fig. 2:**
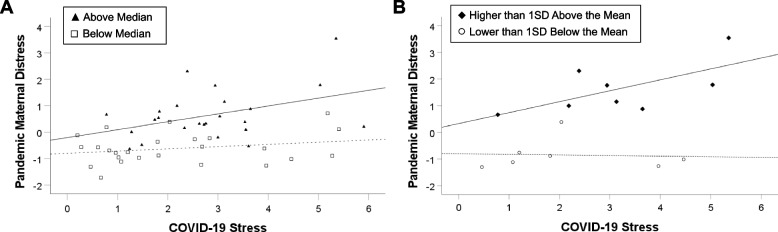
Graphs Representing the Moderating Effect of Pre-pandemic Levels of Maternal Distress on the Relation between COVID-19 Stress and Pandemic Maternal Distress. Note: The scatterplot represents individual data points, and the lines represent the best-fit line for each subgroup. Panel A shows results for the median split, and Panel B shows results for cases which had scores that were greater than one standard deviation above or below the median

Hence, we proceeded to decompose the interaction using the full sample. Amongst those with above-median pre-pandemic distress (N = 24), COVID-19 stress predicted pandemic distress (β = 0.30, *p* = 0.05). However, for those with below-median pre-pandemic distress (N = 25), COVID-19 stress did not predict pandemic distress (β = 0.25, *p* = 0.24; Fig. 2a). Similarly, for mothers with high pre-pandemic distress scores (higher than one standard deviation above the mean, N = 8), COVID-19 stress marginally significantly predicted pandemic distress (β = 0.65, *p* = 0.08). However, for mothers with low pre-pandemic distress scores (lower than one standard deviation below the mean, N = 7), COVID-19 stress did not predict pandemic distress (β = -0.06, *p* = 0.90; Fig. 2b). Hence, this pattern of results shows that for mothers with higher pre-pandemic distress, COVID-19 stress was associated with higher distress during the pandemic. However, for mothers with lower pre-pandemic distress, levels of pandemic maternal distress remained relatively constant regardless of COVID-19 stress.

The same pattern of results remained after maternal education was accounted for as a covariate (see Supplementary Table 2, Additional File 5). Likewise, when impacts to livelihood and household conflict were entered into the model, results remained relatively unchanged. As reported in Additional File 6, a significant interaction was still observed as was a significant main effect of pre-pandemic maternal distress; however, the main effect of COVID-19 stress was no longer significant (see Supplementary Table 5, Additional File 6). Examination of the interaction effects continued to suggest that the relation between COVID-19 stress and pandemic assessed maternal distress was greatest for those with higher levels of pre-pandemic maternal distress.

##### COVID-19 stress and pre-pandemic maternal distress in relation to maternal sensitivity during the pandemic

Table 5 summarizes the results for the model examining maternal sensitivity assessed during the pandemic. Neither of the main effects nor their interaction was significant. The model remained non-significant when maternal sensitivity assessed pre-pandemic and maternal educational level were accounted for as covariates (see Supplementary Table 3, Additional File 4).

**Table 5:** Summary of Regression Analysis Predicting Pandemic Maternal Sensitivity

Block	R^2^	ΔR^2^	*F* Change	β when first entered	β in final model
1. COVID-19 stress Pre-pandemic maternal distress	.019		0.45	.12 .04	.13 .03
2. COVID-19 Stress × Pre-pandemic maternal distress	.023	.004	0.18	.06	.06

^*t*^*p* < .10, * *p* < .05, ** *p* < .01, *** *p* < .001

#### Alternative hypotheses concerning pandemic maternal distress at Wave Two

##### Mediational path

**Fig. 3:**
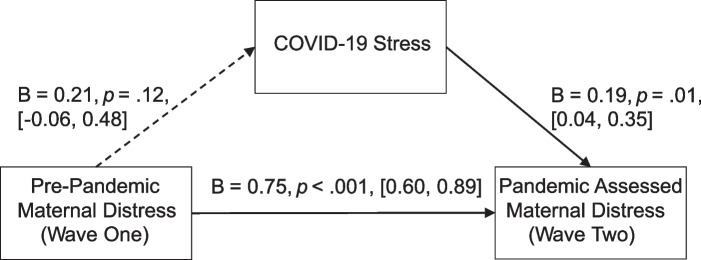
Mediation Model for Maternal Distress. Note: Solid lines represent pathways with confidence intervals consistently above or below zero. Dashed lines represent pathways with confidence intervals that cross 0. Unstandardized estimates are presented as estimate [95% confidence interval]

Figure 3 represents the results of the mediational model, with pre-pandemic maternal distress having an indirect effect on pandemic assessed maternal distress through COVID-19 stress. Pre-pandemic maternal distress predicted maternal distress assessed during the pandemic, Β = 0.75, *p* < 0.001, [0.60, 0.89], but not COVID-19 stress, Β = 0.21, *p* = 0.12, [-0.06, 0.48]. COVID-19 stress predicted concurrent (pandemic assessed) maternal distress in the expected direction Β = 0.19, *p* = 0.01, [0.04, 0.35]. The overall indirect effect was not significant Β = 0.04 [-0.01, 0.13], thus not supporting a mediational model.

##### Prediction of difference scores between pre-pandemic and pandemic maternal distress

**Fig. 4:**
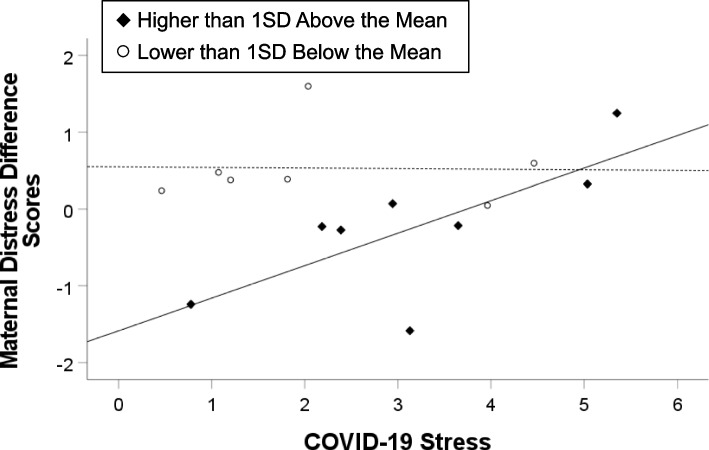
Graph Representing the Moderating Effect of Pre-pandemic Levels of Maternal Distress on the Relation between COVID-19 Stress and Maternal Distress Difference Scores. Note: The scatterplot represents individual data points, and the lines represent the best-fit line for each subgroup

Difference scores were obtained by subtracting the pre-pandemic maternal distress composite score from the pandemic assessed maternal distress composite score. Table 6 summarizes the results for the model examining the difference score as the dependent variable, which were similar to the main analysis. The full model significantly explained 39.0% of the variance in the difference score (*p* < 0.001), and there were significant effects of pre-pandemic maternal distress and COVID-19 stress, as well as their interaction. First, there was an inverse relation between higher levels of pre-pandemic maternal distress and increased distress from pre-pandemic to pandemic (β = -0.52, *p* < 0.001). Second, higher levels of COVID-19 stress were positively associated with increased maternal distress from pre-pandemic to during the pandemic (β = 0.39, *p* < 0.01). Finally, higher levels of pre-pandemic distress in combination with higher levels of COVID-19 stress predicted more positive (i.e., Wave Two > Wave One) change scores in pre-to-pandemic distress (β = 0.37, *p* < 0.01; see Fig. 4). Specifically, for mothers with pre-pandemic distress scores above one standard deviation over the mean, COVID-19 stress significantly predicted pre-to-pandemic maternal distress (β = 0.72, *p* = 0.04). However, for mothers with pre-pandemic distress scores below one standard deviation under the mean, COVID-19 stress did not predict pre-to-pandemic maternal distress (β = -0.02, *p* = 0.96). In sum, while higher levels of pre-pandemic distress associated with less increase in pre-to-pandemic distress, mothers with higher pre-pandemic distress who also experienced higher levels of COVID-19 stress exhibited higher levels of change in pre-to-pandemic distress.

**Table 6:** Summary of Regression Analysis Predicting Maternal Distress Difference Scores

Block	R^2^	ΔR^2^	*F* Change	β when first entered	β in final model
1. COVID-19 stress Pre-pandemic maternal distress	.256		7.92*	.34* -.46**	.39** -.52***
2. COVID-19 stress × Pre-pandemic maternal distress	.390	.134	9.87***	.37**	.37**

^*t*^*p* < .10, * *p* < .05, ** *p* < .01, *** *p* < .001

## Discussion

The purpose of this study was to investigate the interactive effects of concurrent adversity and prior maternal distress on later maternal distress and parenting quality. Specifically, we examined the interactive effect of COVID-19 related adversity and maternal distress assessed prior to the pandemic upon maternal distress and parenting assessed during the pandemic. A composite of maternal distress consisting of psychological distress, parenting stress and trait anxiety was used in this study, while parenting quality was examined using an observational measure of maternal sensitivity. As hypothesized, COVID-19 stress was directly associated with maternal distress assessed during the pandemic, but this was qualified by an interaction effect. Amongst mothers with higher levels of pre-pandemic maternal distress, COVID-19 related stress predicted greater maternal distress assessed during the pandemic, but this pattern was not observed amongst mothers with lower levels of pre-pandemic distress. This effect was robust, after accounting for maternal education, and the interaction remained significant when objective COVID-19 related stressors were considered. Importantly these results extend, but do not contradict, past research indicating that there was less of an increase in mental health symptoms amongst those who had higher levels of pre-pandemic distress [11]. As with other accounts, our examination of difference scores suggested less of an increase (or even, as in our case, a decrease, also see [8]) in distress amongst those with higher pre-pandemic scores. However, by additionally examining the moderating effect of COVID-19 perceived stress we were able to observe that this decline in distress was not the case for mothers who had high levels of pre-pandemic distress *and* felt high levels of COVID-19 stress. In fact, greater amounts of COVID-19 stress were positively associated with higher change scores only amongst mothers with higher levels of pre-pandemic distress. To our knowledge, no other group has examined such an interaction, which may be important in determining the complex way in which past and concurrent perceptions influence mental health.

In contrast, with regards to observed parenting, COVID-19 stress was not associated with maternal sensitivity assessed during the pandemic and there were no interactive effects with pre-pandemic maternal distress, even when pre-pandemic maternal sensitivity and maternal education were considered. Maternal sensitivity assessed during the pandemic was also not associated with maternal distress assessed during the pandemic. Hence, while results support our hypothesis concerning maternal distress, the hypothesis that parenting quality would be differentially affected by the interaction between pre-pandemic maternal distress and COVID-19 stress was not supported.

The current research’s study design helps to rule out a potential confound, present in much research examining the impact of prior mental health factors on the relation between stress and trauma on later functioning. That is, exposure to COVID-19 governmental restrictions was “assigned” to the entire study group – not just those with pre-existing risks – minimizing the likelihood that relations between pre-existing risk and post-adversity outcomes are due to an increased likelihood of risk begetting risk, as opposed to risk leading to greater vulnerability in the face of adversity. Moreover, the current work also considered relations between self-reported perceptions of COVID-19 distress and variation in objective stressors during the same time period and still uncovered a significant interaction between pre-pandemic maternal distress and COVID-19 stress (Additional File 6). Furthermore, to better explain the nature of this association we additionally conducted a mediational analysis examining whether risk begot increased perceptions of risk, as might be expected given research indicating that not only prior mood, but also change in experience during the pandemic, predicts subsequent mood [37]. In contrast to the significant findings concerning the moderating effect of pre-pandemic maternal distress on relations between COVID-19 stress and maternal distress assessed during the pandemic, post-hoc analyses found little support for mediational models. That is, there was no significant evidence that pre-existing distress led to the perception of more pandemic related distress and in turn greater post-pandemic onset distress. Rather, as indicated above, pre-existing distress may interact with subsequent concerns about routine activities (caring for others, housework, finances, hygiene, shopping for provisions, socializing, etc.) to impact subsequent distress. If replicated in a larger study, this would suggest that prevention programs should not only target well-being but also provide practical strategies for dealing with life’s daily hassles as they arise, and/or acknowledge that the skills needed to cope with major stressors and daily life may not always be interchangeable.

Recent research has reported high levels of psychological distress among the general population [7, 38] and parents [6, 39] during the COVID-19 pandemic, and that parents’ levels of depression and anxiety remained relatively consistent before and after the height of the pandemic [12]. The present study extends findings by revealing that while present mental health issues were moderately associated with COVID-19 related stress, within-individual increases in distress were more likely to occur among the mothers of pre-schoolers who had both higher levels of pre-existing distress and greater amounts of perceived COVID-19 stress. This demonstrates that pre-existing levels of risk moderates the extent to which adversity influences subsequent distress, such that those with lower levels of risk are buffered against adversity and are less likely to experience a decline in mental health.

The findings correspond with a previous study that found individuals with greater trait negative affect and detachment, or a history of stressful situations, were more likely to have mental health difficulties during the pandemic [38]. Past local retrospective research has also identified parental age, educational attainment, and parenting values as factors that influenced the extent to which COVID-19 influenced mental health [6]. The current study demonstrates that in addition to the factors above, prior psychological distress, anxiety and parenting stress also predisposes individuals to experience greater mental health distress in response to stress from the pandemic. Our findings align with the notion of individual trajectories in resilience (e.g., resilient, recovering, with chronic symptoms [40, 41]) following potentially traumatic events, with a substantial number of individuals being identified as experiencing chronic symptoms. However, much more than the objective severity of a given stressor (here, the pandemic and associated safety measures), biological and psychological influences seem to play a more important role in one’s individual trajectory towards resilience [42]. Recent evidence showed that individuals who are identified as developing chronic symptoms in response to a stressor have generally been experiencing poorer well-being and higher levels of anxiety [43]. In this context, an implication of the present findings is the importance of promoting and maintaining good mental health, as it may buffer against future difficulties coping with new stressful events as they unfold and enhance resilience at the individual level.

Maintenance of good mental health may be especially important for individuals working in professions that involve exposure to greater risk or high stress environments such as nursing, teaching, or the military. Good prior mental health may enable them to cope with stressful events despite occupational challenges and maintain resilience against developing symptoms of psychological distress. At the same time, however, this work also suggests that preparing individuals for the daily concerns that may arise in such challenging situations can be important, especially for those with higher levels of pre-existing distress. In the context of COVID-19 pandemic, special attention should perhaps be paid to healthcare workers, who experience high levels of psychological distress during and after virus outbreaks [44]. Similarly, this suggests that individuals with good prior mental health may be able to better cope with stressful life events such as divorce, illness, or death of loved ones. As such, social services that work with families who experience stressful events may want to pay attention to existing stressors or prior family functioning. Overall, from a policy perspective, the findings imply that better support should be provided to individuals who have pre-existing mental health difficulties or who are more likely to experience stressors, as they are more vulnerable to experiencing reductions in well-being in response to stressful events, even if their symptoms are not within the clinical range. These results may also suggest the need for routine prevention and support to be offered for those entering high stress fields.

In contrast to findings concerning maternal distress, there was no effect of COVID-19 stress on maternal sensitivity. This is unexpected because parenting quality is expected to change in response to environmental factors. For example, greater support for the primary caregiver led to the greatest improvements in child functioning [45], presumably due to changes in parenting quality. Maternal sensitivity has also been found to vary according to levels of socioeconomic status, parenting stress and internalizing symptoms [18]. However, past local research investigating the effects of COVID-19 found that parents with higher educational levels experienced overall higher levels of emotional well-being, compared to the less educated counterparts [6], as well as less notable decreases in income or cessation of outdoor time [46]. Hence, although our inclusion of relatively highly educated mothers may have decreased variation in the experience of COVID-19 related distress and so made it easier to examine the effects of relatively uniform adversity as a function of past well-being, it may also have decreased the average level of COVID-19 related distress experienced, and so limited our ability to detect any effects on parenting. Indeed, maternal sensitivity in this study was not associated with COVID-19 stress or maternal distress at both timepoints.

Accordingly, a limitation of this study is its small, predominantly Chinese, socioeconomically homogeneous sample, which could have obscured the association between COVID-19 stress and maternal sensitivity. Though we are not aware of any published quantitative accounts, amongst Singapore’s psychiatric community there is a sense that within clinical groups lower socioeconomic status related to greater declines in family functioning during the Circuit Breaker, perhaps in part due to being confined to comparatively smaller spaces with less opportunity to go outdoors (Helen Chen, personal communication, April 15, 2022). In addition, there is some reason to consider that reliance on social support as a buffer against stress may vary by ethnicity [47, 48], further limiting the generalizabilty of our findings, especially since COVID-19 restrictions intentionally limited social gatherings.

Second, it is possible that although all participants were exposed to the COVID-19 pandemic and its associated restrictions, individuals had unique experiences in their exposure to specific COVID-19 stressors that could have influenced levels of perceived stress from COVID-19. Still, analyses incorporating additional data about the COVID-19 experiences continued to reveal a significant interaction between pre-pandemic distress and perceptions of COVID-19 stress, though post-hoc tests were no longer significant (see Additional File 6). If possible, future research should account for objective sources of stress that correspond to areas in which participants rated perceived stress, to reduce the likelihood that higher perceived stress stems from increased exposure to a greater number of domain relevant stressors. The current research group will be following up with larger Singaporean cohorts in order to answer some of these questions, as well as to follow up on how COVID-19 experiences may influence future child development.

Finally, our questionnaire considered levels of COVID-19 stress at three different time periods. Mothers were asked to retrospectively report on COVID-19 related stress for the first two time periods and to consider recent and concurrent COVID-19 related stress for the third. Thus, our questionnaire may have been subject to memory bias [49]. Indeed, emotional state and perceptions concerning the passage of time during the COVID-19 pandemic may be linked [50]. We therefore conducted post-hoc analyses examining the potential association between the amount of time elapsed between the Circuit Breaker and questionnaire administration and COVID-19 stress at each time period (i.e., February to March 2020, April to May 2020, June 2020 onwards), as well as with overall COVID-19 stress and maternal distress assessed during the pandemic. We did not uncover significant associations. This, coupled with the fact that the various restriction phases in Singapore were clearly titled (e.g., Circuit Breaker, Phase One) defined specific group size limits [51], and were clearly communicated to the public via television and social media channels, suggests that memory biases may not have played a large role in explaining post-pandemic onset distress (see Additional File 8). Still, questionnaires prospectively querying stress at each phase may have been able to deepen our understanding of how interactions between past- and present- mental health and perceived stress unfold.

## Conclusions

In summary, this study demonstrates the differential effects of COVID-19 stress on mental health. In conjunction with existent literature, this work indicates that the impact of adversity upon mental health is not uniform across all individuals, hence special support should be provided to individuals with existing difficulties who are also at risk for encountering future adversity, and address both basic mental health as well as situation relevant coping strategies.

## Supplementary Information

**Additional file 1:** COVID-19 Situation in Singapore. A description of COVID-19 illness prevalence and restrictions in Singapore.

**Additional file 2:** COVID-19 Phases within the COVID-19 Questionnaire. A description of the three phases of the Pandemic in Singapore: COVID-19’s Beginning, the Circuit Breaker (Lockdown), and Post-Circuit Breaker.

**Additional file 3:** Comparisons Between Mothers Who Completed the First Visit Only vs. Both Visits. A description of the tests comparing both groups of mothers and the results, including Supplementary Table 1.

**Additionalfile 4:** COVID-19 Questionnaire. A copy of the questionnaire used to assess stress and stressors related to COVID-19.

**Additional file 5:** Moderation Analyses Controlling for Covariates. A description of the moderation analyses with covariates, including Supplementary Tables 2 and 3.

**Additional file 6:** Secondary Analyses of Objective COVID-19 Experiences. A description of the measures and of objective COVID-19 experiences and secondary analyses controlling for them, including Supplementary Tables 4 and 5.

**Additional file 7:** Results of Moderation Analyses Without Outliers. Results of the main moderation analyses for pandemic maternal distress without three outliers, and Supplementary Table 6.

**Additional file 8:** Post-hoc Analyses Examining Potential Memory Bias. A description of analyses to investigate whether retrospective reports of COVID-19 stress could have biased results.

## Data Availability

The deidentified datasets used and/or analysed during the current study are available from the corresponding author on reasonable request. Access to video data (which is inherently not de-identified) may be limited to comply with local laws, IRB policies, and out of respect for participants' privacy.

## Abbreviations

PTSD: Posttraumatic stress disorder
SPACE: Singapore Parenting And Cognition in Early childhood
K10: Kessler Psychological Distress Scale
PSI: Parenting Stress Index
PD: Parental Dysfunction
P-CDI: Parent-Child Dysfunctional Interaction
DC: Difficult Child
STAI: State-Trait Anxiety Inventory
