# Supplementary material for: Maternal distress and parenting during COVID-19: differential effects related to pre-pandemic distress?
Source: BMC Psychiatry. 2023 May 29;23:374. doi: 10.1186/s12888-023-04867-w (PMC10225758; doi:10.1186/s12888-023-04867-w)
Supplement: Supplementary file 1 — Additional file 1: COVID-19 Situation in Singapore. A description of COVID-19 illness prevalence and restrictions in Singapore. [file 12888_2023_4867_MOESM1_ESM.docx]

**COVID-19 Situation in Singapore**

In Singapore, cases of COVID-19 illness were relatively well-controlled, with low infection and death rates. According to Singapore’s Ministry of Health [1], in the two weeks preceding the last date of the current work’s data collection (i.e., January 9^th^ 2021), COVID-19 had been detected in only 0.04% of the “community” and 1.03% of all people residing in Singapore (i.e., community cases plus cases that were brought into Singapore from abroad and/or observed in dormitories housing migrant workers, with restricted access to the larger community). Similarly, as of January 9^th^ 2021, the cumulative number of all COVID-19 deaths, within Singapore was listed as twenty-nine, which roughly translates to 0.000005% of all people residing in Singapore at that time.

COVID-19 restrictions in Singapore were restrictive and closely followed, which likely had substantial impact on residents. During the height of the restrictions (April 7^th^ – June 1^st^ 2020) other than essential workers, people were not allowed out of their homes except to exercise and go to the grocery store, with any such outings limited to one person at a time. As another example, from June 1^st^ 2020 until March 29^th^ 2022 there were limits on the number of daily social interactions allowed and the number of daily allowable guests per household, ranging from 0-8 depending on hospitalization and infection rates. Because the majority (95%) of Singaporeans do not live in landed homes [2], it is likely that most people did not have yards or significant personal outdoor space, and so felt the impact of limits on outdoor life; although it is of note that the longer-term impact on outdoor time was felt to a greater extent in lower income Singaporean families [3]. From April 14^th^ 2020 to March 29^th^ 2022 masks were compulsory indoors and outdoors, except during vigorous exercise. Noncompliance with regulations could be identified via, e.g., “Safe Distancing Ambassadors” and there was extensive virtual tracking via apps or digital tokens. Noncompliance could be met with fines and/or imprisonment.

References

1. Ministry of Health Singapore: **09 January 2021 daily report on COVID-19**. In: *COVID-19 situation report.* 2021.

2. Singapore Department of Statistics: **Households**. In: *Households - latest data.* Singapore; 2021.

3. Sum KK, Cai S, Law E, Cheon B, Tan G, Loo E, Lee YS, Yap F, Chan JKY, Daniel M *et al*: **COVID-19–related life experiences, outdoor play, and long-term adiposity changes among preschool- and school-aged children in Singapore 1 year after lockdown**. *JAMA Pediatrics* 2022, **176**(3):280.
