# Supplementary material for: Maternal distress and parenting during COVID-19: differential effects related to pre-pandemic distress?
Source: BMC Psychiatry. 2023 May 29;23:374. doi: 10.1186/s12888-023-04867-w (PMC10225758; doi:10.1186/s12888-023-04867-w)
Supplement: Supplementary file 2 — Additional file 2: COVID-19 Phases within the COVID-19 Questionnaire. A description of the three phases of the Pandemic in Singapore: COVID-19’s Beginning, the Circuit Breaker (Lockdown), and Post-Circuit Breaker. [file 12888_2023_4867_MOESM2_ESM.docx]

**COVID-19 Phases Within the COVID-19 Questionnaire**

For each item in the COVID-19 questionnaire, participants were asked to give separate ratings for three phases of the Pandemic: COVID-19’s Beginning, the Circuit Breaker (Lockdown), and Post-Circuit Breaker.

The *Beginning* period refers to February-March 2020. Singapore’s first imported case was reported on 23^rd^ January 2020, and local transmission was first reported on 4^th^ February 2020 [1]. Between February to early March 2020, local transmission remained low, and infection control measures mainly included travel restrictions, quarantine orders for inbound visitors and contact tracing, and some additional safe distancing precautions [2].

The *Circuit Breaker* refers to April and May of 2020. Infections rose to above 1,000 in March and April 2020 [3]. In order to curb escalating infections, a nationwide lockdown was imposed from 7^th^ April 2020 to 1^st^ June 2020 [4]. All workplaces, schools, and businesses were ordered to close or adopt home-based arrangements, except for those that provided essential services or operated in essential sectors. Social gatherings and visits were disallowed, and all residents were required to stay home except to carry out essential activities [5, 6] (see [7] for a summary of events leading up to and describing the lockdown measures).

*Post Circuit-Breaker* refers to the period starting in June 2020. As infections within the community stabilized and cases among migrant worker populations declined, restrictions were slowly eased [8]. From 2^nd^ June 2020, workplaces, schools, and most businesses were gradually reopened, and social gatherings were allowed, though with safeguards in place [9, 10], such as limiting social gatherings to groups of two, five, or for a short period of time, eight people, and limiting the number of social gatherings per day to one. Measures affecting the community’s daily lives were largely stable, though with incremental relaxations of permissible activities (e.g., restaurants opened to small groups with social distancing), though activities such as singing and playing music in restaurants remained forbidden, from June 2020 to 9^th^ January 2021 when data collection was completed [11]. In general, this period represents a time when economic and social activities were progressively allowed to resume with safe distancing measures in place [12].

References

1. **COVID-19 situation report** [<https://covidsitrep.moh.gov.sg/>]

2. Lee VJ, Chiew CJ, Khong WX: **Interrupting transmission of COVID-19: Lessons from containment efforts in Singapore**. *Journal of Travel Medicine* 2020, **27**(3).

3. Lai L: **Singapore to enter phase 3 on Dec 28: Groups of 8 to be allowed for social gatherings**. In: *The Straits Times.* Singapore; 2020.

4. Cheong D: **Coronavirus: Most workplaces to close, schools will move to full home-based learning from next week, says PM Lee**. In: *The Straits Times.* Singapore; 2020.

5. **Circuit Breaker to minimise further spread of COVID-19** [<https://www.moh.gov.sg/news-highlights/details/circuit-breaker-to-minimise-further-spread-of-covid-19>]

6. **Easing the tighter Circuit Breaker measures, preparing for gradual resumption of activity after 1 June** [<https://www.moh.gov.sg/news-highlights/details/easing-the-tighter-circuit-breaker-measures-preparing-for-gradual-resumption-of-activity-after-1-june>]

7. Jacinta I, Chen P, Yap JC-H, Hsu LY, Teo YY: **COVID-19 and Singapore: From early response to circuit breaker**. *Annals, Academy of Medicine, Singapore* 2020, **49**:561-572.

8. Sin Y: **Coronavirus: S'pore to start phase 2 reopening on June 19; F&B dine-in, social gatherings of up to 5 people allowed**. In: *The Straits Times.* Singapore; 2020.

9. **Safe Re-opening: How Singapore will resume activities after the circuit breaker** [<https://www.gov.sg/article/safe-re-opening-how-singapore-will-resume-activities-after-the-circuit-breaker>]

10. **Post-circuit breaker – when can we move on to phases 2 and 3?** [<https://www.gov.sg/article/post-circuit-breaker-when-can-we-move-on-to-phases-2-and-3>]

11. **Moving into Phase 3 of Re-Opening on 28 Dec 2020** [<https://www.gov.sg/article/moving-into-phase-3-of-re-opening-on-28-dec-2020>]

12. **Moving into Phase 2: What activities can resume** [<https://www.gov.sg/article/moving-into-phase-2-what-activities-can-resume>]
