# Supplementary material for: Maternal distress and parenting during COVID-19: differential effects related to pre-pandemic distress?
Source: BMC Psychiatry. 2023 May 29;23:374. doi: 10.1186/s12888-023-04867-w (PMC10225758; doi:10.1186/s12888-023-04867-w)
Supplement: Supplementary file 3 — Additional file 3: Comparisons Between Mothers Who Completed the First Visit Only vs. Both Visits. A description of the tests comparing both groups of mothers and the results, including Supplementary Table 1. [file 12888_2023_4867_MOESM3_ESM.docx]

**Comparisons Between Mothers Who Completed the First Visit Only vs. Both Visits**

Of the 67 participants that took part in the first visit, 16 (37.5% boys) attended one visit only and 51 (51.0% boys) attended both visits. A chi-square test of homogeneity revealed no differences in proportions of child gender (*p* = .35), and independent sample t-tests were conducted to investigate differences in maternal education, maternal year of birth, child age, child gender, as well as baseline maternal distress and observed maternal sensitivity. There were no significant differences between mothers who completed one visit only versus those who completed both visits (see Supplementary Table 1).

Supplementary Table 1

*Independent Sample T-test Results Comparing Scores of Mothers Who Completed the First Visit Only vs. Both Visits*

|  | First Visit Only | | Both Visits | |  |  |  |
| --- | --- | --- | --- | --- | --- | --- | --- |
|  | Mean | SD | Mean | SD | *t* | df | *p* |
| Maternal education | 3.7 | 0.9 | 4.0 | 0.8 | -1.07 | 64 | .29 |
| Maternal year of birth | 1983.8 | 4.5 | 1983.5 | 4.2 | 0.24 | 65 | .81 |
| Child age | 4.5 | 0.2 | 4.5 | 0.3 | 0.40 | 65 | .69 |
| Maternal distress | -0.1 | 0.8 | 0.0 | 1.1 | -0.21 | 63 | .42 |
| Maternal sensitivity | 0.4 | 0.2 | 0.4 | 0.2 | 0.50 | 63 | .62 |
