# Supplementary material for: Maternal distress and parenting during COVID-19: differential effects related to pre-pandemic distress?
Source: BMC Psychiatry. 2023 May 29;23:374. doi: 10.1186/s12888-023-04867-w (PMC10225758; doi:10.1186/s12888-023-04867-w)
Supplement: Supplementary file 4 — Additionalfile 4: COVID-19 Questionnaire. A copy of the questionnaire used to assess stress and stressors related to COVID-19. [file 12888_2023_4867_MOESM4_ESM.docx]

**COVID-19 Questionnaire**

**COVID-19 Stress**

On a scale of 0 to 10, please type in a number to indicate how much COVID-19 affected *your* stress levels for the following items.
Please answer separately with regard to **beginning of COVID-19 (Feb-Mar 2020), during the circuit breaker (Apr-May 2020), and post circuit breaker (Jun 2020 - current)?** 0: not stressful at all; 10: extremely stressful;
 If not applicable, please select "Not Applicable"
 *(e.g., if there are no elderly people in the household, you may select "Not Applicable" for the statement "stress caring for elderly people" )*

|  | **Feb-Mar** | **Apr-May** | **Jun-*current*** |
| --- | --- | --- | --- |
| Stress caring for children | ▼ 0- Not stressful at all (1 ... Not Applicable (12) | ▼ 0- Not stressful at all (1 ... Not Applicable (12) | ▼ 0- Not stressful at all (1 ... Not Applicable (12) |
| Stress caring for elderly people | ▼ 0- Not stressful at all (1 ... Not Applicable (12) | ▼ 0- Not stressful at all (1 ... Not Applicable (12) | ▼ 0- Not stressful at all (1 ... Not Applicable (12) |
| Stress about housework | ▼ 0- Not stressful at all (1 ... Not Applicable (12) | ▼ 0- Not stressful at all (1 ... Not Applicable (12) | ▼ 0- Not stressful at all (1 ... Not Applicable (12) |
| Stress from job/business demands | ▼ 0- Not stressful at all (1 ... Not Applicable (12) | ▼ 0- Not stressful at all (1 ... Not Applicable (12) | ▼ 0- Not stressful at all (1 ... Not Applicable (12) |
| Stress relating to inconveniences in working/doing business (e.g. technical issues related to working remotely) | ▼ 0- Not stressful at all (1 ... Not Applicable (12) | ▼ 0- Not stressful at all (1 ... Not Applicable (12) | ▼ 0- Not stressful at all (1 ... Not Applicable (12) |
| Stress about losing job/business | ▼ 0- Not stressful at all (1 ... Not Applicable (12) | ▼ 0- Not stressful at all (1 ... Not Applicable (12) | ▼ 0- Not stressful at all (1 ... Not Applicable (12) |
| Stress about financial difficulties | ▼ 0- Not stressful at all (1 ... Not Applicable (12) | ▼ 0- Not stressful at all (1 ... Not Applicable (12) | ▼ 0- Not stressful at all (1 ... Not Applicable (12) |
| Stress from difficulties in getting necessities (e.g. protection gears, medicine, groceries, computers) | ▼ 0- Not stressful at all (1 ... Not Applicable (12) | ▼ 0- Not stressful at all (1 ... Not Applicable (12) | ▼ 0- Not stressful at all (1 ... Not Applicable (12) |
| Stress from suboptimal living conditions (e.g. overcrowding, repair work being delayed) | ▼ 0- Not stressful at all (1 ... Not Applicable (12) | ▼ 0- Not stressful at all (1 ... Not Applicable (12) | ▼ 0- Not stressful at all (1 ... Not Applicable (12) |
| Stress about own health and safety | ▼ 0- Not stressful at all (1 ... Not Applicable (12) | ▼ 0- Not stressful at all (1 ... Not Applicable (12) | ▼ 0- Not stressful at all (1 ... Not Applicable (12) |
| Stress about family members' health and safety | ▼ 0- Not stressful at all (1 ... Not Applicable (12) | ▼ 0- Not stressful at all (1 ... Not Applicable (12) | ▼ 0- Not stressful at all (1 ... Not Applicable (12) |
| Stress from reading about negative news | ▼ 0- Not stressful at all (1 ... Not Applicable (12) | ▼ 0- Not stressful at all (1 ... Not Applicable (12) | ▼ 0- Not stressful at all (1 ... Not Applicable (12) |
| Stress from limited opportunities to travel or go outside | ▼ 0- Not stressful at all (1 ... Not Applicable (12) | ▼ 0- Not stressful at all (1 ... Not Applicable (12) | ▼ 0- Not stressful at all (1 ... Not Applicable (12) |
| Stress from limited opportunities to socialize with friends/colleagues | ▼ 0- Not stressful at all (1 ... Not Applicable (12) | ▼ 0- Not stressful at all (1 ... Not Applicable (12) | ▼ 0- Not stressful at all (1 ... Not Applicable (12) |
| Stress from limited opportunities to visit family/relatives | ▼ 0- Not stressful at all (1 ... Not Applicable (12) | ▼ 0- Not stressful at all (1 ... Not Applicable (12) | ▼ 0- Not stressful at all (1 ... Not Applicable (12) |
| Stress concerning own hygiene practices (e.g., washing hands) | ▼ 0- Not stressful at all (1 ... Not Applicable (12) | ▼ 0- Not stressful at all (1 ... Not Applicable (12) | ▼ 0- Not stressful at all (1 ... Not Applicable (12) |
| Stress concerning my children’s hygiene practices (e.g., washing hands) | ▼ 0- Not stressful at all (1 ... Not Applicable (12) | ▼ 0- Not stressful at all (1 ... Not Applicable (12) | ▼ 0- Not stressful at all (1 ... Not Applicable (12) |
| Other major stressors not listed here, please specify: | ▼ 0- Not stressful at all (1 ... Not Applicable (12) | ▼ 0- Not stressful at all (1 ... Not Applicable (12) | ▼ 0- Not stressful at all (1 ... Not Applicable (12) |

**Impact on Livelihood**

**How much do you think Covid-19 impacted your livelihood beginning of COVID-19 (Feb-Mar 2020), during the circuit breaker (Apr-May 2020), and post circuit breaker (Jun 2020 - current)?**
4- Very large negative impact; 3-Relatively large negative impact; 2-Some negative impact; 1-No negative impact

|  | **Feb-Mar** | **Apr-May** | **Jun-*current*** |
| --- | --- | --- | --- |
| Impact on **YOUR** livelihood | ▼ 4. Very large negative impact (1 ... 1. No negative impact (4) | ▼ 4. Very large negative impact (1 ... 1. No negative impact (4) | ▼ 4. Very large negative impact (1 ... 1. No negative impact (4) |
| Impact on **SPACE Child's Father's** livelihood | ▼ 4. Very large negative impact (1 ... 1. No negative impact (4) | ▼ 4. Very large negative impact (1 ... 1. No negative impact (4) | ▼ 4. Very large negative impact (1 ... 1. No negative impact (4) |

**Conflict with Household Members**

**On a scale of 0 to 10, please type in a number to indicate how much *you*felt, experienced, or did the following, beginning of COVID-19 (Feb-Mar 2020), during the circuit breaker (Apr-May 2020), and post circuit breaker (Jun 2020 - current)?**0: never; 10: always;
If not applicable, please select "Not Applicable" 
*(e.g., if the only children in the household are infants, select "Not Applicable" for the questions about children)*

|  | **Feb-Mar** | **Apr-May** | **Jun-*current*** |
| --- | --- | --- | --- |
| Conflicted with children | ▼ 0- Never (1 ... Not Applicable (12) | ▼ 0- Never (1 ... Not Applicable (12) | ▼ 0- Never (1 ... Not Applicable (12) |
| Conflicted with spouse | ▼ 0- Never (1 ... Not Applicable (12) | ▼ 0- Never (1 ... Not Applicable (12) | ▼ 0- Never (1 ... Not Applicable (12) |
| Conflicted with domestic helper | ▼ 0- Never (1 ... Not Applicable (12) | ▼ 0- Never (1 ... Not Applicable (12) | ▼ 0- Never (1 ... Not Applicable (12) |
| Conflicted with other members of the family | ▼ 0- Never (1 ... Not Applicable (12) | ▼ 0- Never (1 ... Not Applicable (12) | ▼ 0- Never (1 ... Not Applicable (12) |

**COVID-19 Media Exposure**

How much exposure to the COVID-19 pandemic do you/did you have through watching television, the internet, radio, newspaper or magazines, or other forms of media?
Please answer separately with regard to **beginning of COVID-19 (Feb-Mar 2020), during the circuit breaker (Apr-May 2020), and post circuit breaker (Jun 2020 - current)?**
 (4-Very Frequent, 3-Often, 2-Some, 1-No exposure)

|  | **Feb-Mar** | **Apr-May** | **Jun-*current*** |
| --- | --- | --- | --- |
| Television | ▼ 4-Very frequent (1 ... 1-No exposure (4) | ▼ 4-Very frequent (1 ... 1-No exposure (4) | ▼ 4-Very frequent (1 ... 1-No exposure (4) |
| Internet | ▼ 4-Very frequent (1 ... 1-No exposure (4) | ▼ 4-Very frequent (1 ... 1-No exposure (4) | ▼ 4-Very frequent (1 ... 1-No exposure (4) |
| Radio | ▼ 4-Very frequent (1 ... 1-No exposure (4) | ▼ 4-Very frequent (1 ... 1-No exposure (4) | ▼ 4-Very frequent (1 ... 1-No exposure (4) |
| Newspaper or magazines | ▼ 4-Very frequent (1 ... 1-No exposure (4) | ▼ 4-Very frequent (1 ... 1-No exposure (4) | ▼ 4-Very frequent (1 ... 1-No exposure (4) |
| Other forms of media, please specify: | ▼ 4-Very frequent (1 ... 1-No exposure (4) | ▼ 4-Very frequent (1 ... 1-No exposure (4) | ▼ 4-Very frequent (1 ... 1-No exposure (4) |
