# Supplementary material for: Maternal distress and parenting during COVID-19: differential effects related to pre-pandemic distress?
Source: BMC Psychiatry. 2023 May 29;23:374. doi: 10.1186/s12888-023-04867-w (PMC10225758; doi:10.1186/s12888-023-04867-w)
Supplement: Supplementary file 5 — Additional file 5: Moderation Analyses Controlling for Covariates. A description of the moderation analyses with covariates, including Supplementary Tables 2 and 3. [file 12888_2023_4867_MOESM5_ESM.docx]

**Moderation Analyses Controlling for Covariates**

We repeated the analyses with maternal education as a covariate. Maternal education and/or maternal sensitivity was added in the first block, COVID-19 stress and pre-pandemic maternal distress were added in the second block, and the interaction term in the third block.

**Results for Pandemic Maternal Distress as the Outcome Variable**

Maternal education was not a significant predictor of maternal distress assessed during the pandemic when first entered (β = -.11, *p* = .44) and in the final model (β = .04, *p* = .62; see Supplementary Table 2), and the same pattern of results as the main findings were observed.

**Supplementary Table 2**

*Summary of Regression Analysis Predicting Pandemic Maternal Distress at Wave Two Controlling for Maternal Education*

| Block | R^2^ | ΔR^2^ | *F* Change | β when first entered | β in final model |
| --- | --- | --- | --- | --- | --- |
| 1. Maternal Education | .013 |  | .61 | -.11 | .04 |
| 1. COVID-19 stress Pre-pandemic maternal distress | .749 | .736 | 66.09*** | .20*  .81*** | .23**  .77*** |
| 1. COVID-19 stress × Pre-pandemic maternal distress | .793 | .043 | 9.20*** | .21** | .21** |
|  |  |  |  |  |  |

*^t^ p* < .10, * *p* < .05, ** *p* < .01, *** *p* < .001

**Results for Pandemic Maternal Sensitivity as the Outcome Variable**

A summary of the results can be found in Supplementary Table 3. Maternal education was not a significant predictor of maternal sensitivity assessed during the pandemic when first entered (β = -.07, *p* = .51) and in the final model (β = -.09, *p* = .46). Expectedly, pre-pandemic maternal sensitivity was a significant predictor of pandemic maternal sensitivity when first entered (β = .65, *p* < .001) and in the final model (β = .65, *p* < .001). However, the same overall pattern of non-significant results remained.

**Supplementary Table 3**

*Summary of Regression Analysis Predicting Pandemic Maternal Sensitivity at Wave Two Controlling for Maternal Education and Pre-pandemic Maternal Sensitivity*

| Block | R^2^ | ΔR^2^ | *F* Change | β when first entered | β in final model |
| --- | --- | --- | --- | --- | --- |
| 1. Maternal education   Pre-pandemic maternal  sensitivity | .435 |  | 17.29*** | -.07  .65*** | -.09  .65*** |
| 1. COVID-19 stress Pre-pandemic maternal distress | .439 | .004 | 0.16 | -.06  .05 | -.05  .04 |
| 1. COVID-19 stress × Pre-pandemic maternal distress | .448 | .009 | 0.70 | .10 | .10 |
|  |  |  |  |  |  |

*^t^ p* < .10, * *p* < .05, ** *p* < .01, *** *p* < .001
