# Supplementary material for: Maternal distress and parenting during COVID-19: differential effects related to pre-pandemic distress?
Source: BMC Psychiatry. 2023 May 29;23:374. doi: 10.1186/s12888-023-04867-w (PMC10225758; doi:10.1186/s12888-023-04867-w)
Supplement: Supplementary file 6 — Additional file 6: Secondary Analyses of Objective COVID-19 Experiences. A description of the measures and of objective COVID-19 experiences and secondary analyses controlling for them, including Supplementary Tables 4 and 5. [file 12888_2023_4867_MOESM6_ESM.docx]

**Secondary Analyses of Objective COVID-19 Experiences**

**Measures**

Participants were asked about potentially stressful experiences related to COVID-19 within the COVID-19 questionnaire (see Additional File 3 for the questionnaire).

**COVID-19 Diagnoses and Isolation Orders.** Participants were asked if any household members, people in their neighbourhood, or who were important to them or whom they saw regularly had been diagnosed with COVID-19 or received a Quarantine Order, Leave of Absence or Stay-Home Notice. Participants were coded as “1” if they indicated “yes” to any of these questions, and “0” if they indicated “no” for all questions.

**Impact on Livelihood.** Participants were asked to separately rate how much COVID-19 impact their and their co-parents’ livelihoods, from *no negative impact* (1) to *very large negative impact* (4). Items were averaged to create a livelihood score (α = .85).

**Conflict with Household Members.** Participants were asked to separately rate how much they felt conflicted with their children, spouse, domestic helper, and other members of the family from *never* (0) to *always* (10). Responses of *Not Applicable* were given a score of zero, and items were averaged to create a score representing overall conflict with household members (α = .90).

**COVID-19 Media Exposure.** Participants were asked to separately rate how much exposure they had to COVID-19 pandemic through the television, internet, radio, newspaper or magazines, and other forms of media from *no exposure* (1) to *very frequent* (4). Items were averaged to create a total media exposure score (α = .78).

**Results**

**Statistical Plan.** First, an independent-samples t-test was conducted to analyse if participants who knew someone who had received a COVID-19 diagnosis or isolation order (N = 10) had COVID-19 stress and maternal distress scores that differed from those who did not (N = 40). Second, relationships among COVID-19 stress, maternal distress at both timepoints and COVID-19 experiences were examined using simple bivariate corelations. Third, variables that were significantly related to either COVID-19 stress and maternal distress were added as covariates in the main moderation analyses.

**Independent-samples T-test.** The COVID-19 stress scores of participants who knew someone who had received a COVID-19 diagnosis or isolation order (2.89 ± 1.23) were higher than those who did not (2.41 ± 1.57), though this difference was not significant *t*(48) = .90, *p* = .37. For pre-pandemic maternal distress, a Welch t-test was run instead as the assumption of homogeneity of variances was violated. The scores of participants who knew someone who had received a COVID-19 diagnosis or isolation order (0.65 ± 1.44) were higher than those who did not (-0.17 ± 0.90), though this difference was not significant *t*(10.81) = 1.73, *p* = .11. Similarly for maternal distress during the pandemic, the scores of participants who knew someone who had received a COVID-19 diagnosis or isolation order (0.43 ± 1.25) were higher than those who did not (-0.12 ± 0.92), though this difference was not significant *t*(46) = 1.54, *p* = .13. Hence, knowledge of someone who had been diagnosed with COVID-19 or received an isolation order did not influence COVID-19 stress or maternal distress scores and was not included in subsequent analyses.

**Correlations Between COVID-19 Experiences, COVID-19 Stress and Maternal Distress.** The Pearson correlations between these variables are represented in Supplemental Table 4. Livelihood impact significantly correlated with COVID-19 stress (*r* = .47, *p* < .001) and conflict with household members significantly correlated with COVID-19 stress (*r* = .58, *p* < .001), pre-pandemic maternal distress (*r* = .31, *p* = .03) and maternal distress during the pandemic (*r* = .48, *p* < .001). Hence, the main analyses were repeated with livelihood impact and conflict with household members included as covariates.

**Supplemental Table 4**

*Pearson Correlations Between Overall Scores of COVID-19 Stress and COVID-19 Experiences*

|  | Livelihood impact | Conflict with household members | COVID-19 media exposure |
| --- | --- | --- | --- |
| COVID-19 stress | .47** | .58*** | .11 |
| Pre-pandemic maternal distress (Wave One) | -.06 | .31* | -.12 |
| Pandemic maternal distress (Wave Two) | .07 | .48*** | -.12 |

*^t^ p* < .10, * *p* < .05, ** *p* < .01, *** *p* < .001

**Main Analyses With COVID-19 Covariates.** The regression analysis examining the moderating effect of prior maternal distress on the relationship between COVID-19 stress and later maternal distress was repeated, with livelihood impact and conflict with household members examined as covariates within the same model. Supplemental Table 5 summarizes the results. Although conflict with household members significantly predicted pandemic maternal distress when first entered into the model (β = .49, *p* < .001), its effect became non-significant in the final regression equation (β = .12, *p* = .18). On the other hand, livelihood impact did not significantly predict later maternal distress when first entered into the model (β = -.05, *p* = .72), though it was a marginally significant predictor in the final model (β = .13, *p* < .10).

With the addition of the covariates, in the final model, the main effect of pre-pandemic maternal distress continued to be significant (β = .77, *p* < .001), although the main effect of COVID-19 stress was no longer significant (β = .10, *p* = .31). However, the interaction term between pre-pandemic maternal distress and COVID-19 stress continued to be significant (β = .20, *p* < .01). Hence, the moderating effect of prior maternal distress on the relationship between COVID-19 stress and later maternal distress was still observed even when objective COVID-19 experiences were accounted for.

Further examination of the interaction effect revealed a similar trend to the main findings, though they did not reach significance. For mothers with above-median levels of pre-pandemic distress, COVID-19 stress did not predict pandemic maternal distress (β = .29, *p* = .26), though results were in the expected direction. As expected, COVID-19 stress did not predict pandemic maternal distress for mothers with below-median levels of pre-pandemic distress (β = -.10, *p* = .70). However, conflict with household members significantly predicted pandemic maternal distress (β = .63, *p* < .01). Similarly, for cases with pre-pandemic maternal distress scores higher than one standard deviation above the mean, COVID-19 stress did not predict pandemic maternal distress (β = .58, *p* = .32), though the results were again in the expected direction. Again, as expected for cases with pre-pandemic maternal distress scores lower than one standard deviation below the mean, COVID-19 stress did not predict pandemic maternal distress (β = -.03, *p* = .93). However, conflict with household members significantly predicted pandemic maternal distress (β = 1.10, *p* = .04). This suggests that mothers with low pre-pandemic distress were more likely to have conflicts with household members during the COVID-19 pandemic, which contributed to higher concurrent levels of distress.

**Supplemental Table 5**

*Summary of Regression Analysis Predicting Pandemic-Assessed Maternal Distress with Livelihood Impact and Conflict with Household Members As Covariates*

| Block | R^2^ | ΔR^2^ | *F* Change | β when first entered | β in final model |
| --- | --- | --- | --- | --- | --- |
| 1. Livelihood impact Conflict with household members | .235 |  | 7.08** | -.05  .49** | .13^t^  .12 |
| 1. COVID-19 stress Pre-pandemic maternal distress | .776 | .541 | 53.24*** | .04  .79*** | .10  .77*** |
| 1. COVID-19 Stress × Pre-pandemic maternal distress | .813 | .037 | 8.44** | .20** | .20** |

*^t^ p* < .10, * *p* < .05, ** *p* < .01, *** *p* < .001
