# Supplementary material for: Maternal distress and parenting during COVID-19: differential effects related to pre-pandemic distress?
Source: BMC Psychiatry. 2023 May 29;23:374. doi: 10.1186/s12888-023-04867-w (PMC10225758; doi:10.1186/s12888-023-04867-w)
Supplement: Supplementary file 7 — Additional file 7: Results of Moderation Analyses Without Outliers. Results of the main moderation analyses for pandemic maternal distress without three outliers, and Supplementary Table 6. [file 12888_2023_4867_MOESM7_ESM.docx]

**Results of Moderation Analyses Without Outliers**

The results of the moderation analyses without three cases with possible undue influence are summarized in Supplementary Table 6. Most notably, the interaction effect was marginally significant (β = .18, *p* = .052).

**Supplementary Table 6**

*Summary of Regression Analysis Predicting Pandemic Maternal Distress Without Outliers*

| Block | R^2^ | ΔR^2^ | *F* Change | β when first entered | β in final model |
| --- | --- | --- | --- | --- | --- |
| 1. COVID-19 stress Pre-pandemic maternal distress | .683 |  | 46.26*** | . 19*  .78*** | .25**  .79*** |
| 1. COVID-19 stress × Pre-pandemic maternal distress | .710 | .027 | 3.99^t^ | .18^t^ | .18^t^ |
|  |  |  |  |  |  |

*^t^ p* < .10, * *p* < .05, ** *p* < .01, *** *p* < .001
