# Supplementary material for: Maternal distress and parenting during COVID-19: differential effects related to pre-pandemic distress?
Source: BMC Psychiatry. 2023 May 29;23:374. doi: 10.1186/s12888-023-04867-w (PMC10225758; doi:10.1186/s12888-023-04867-w)
Supplement: Supplementary file 8 — Additional file 8: Post-hoc Analyses Examining Potential Memory Bias. A description of analyses to investigate whether retrospective reports of COVID-19 stress could have biased results. [file 12888_2023_4867_MOESM8_ESM.docx]

**Post-hoc Analyses Examining Potential Memory Bias**

The number of days between June 2020 and the date of the Wave Two visit was calculated. There were no significant correlations between this number and average COVID-19 stress (*r* = .15, *p* = .28) or pandemic assessed maternal distress (*r* = .01, *p* = .96) used in the main analyses, as well as with the COVID-19 stress scores for each time period (*r* = .09 to .22, *p* > .05). This suggests that the timing of the Wave Two visit during the pandemic was not related to COVID-19 stress scores or self-reports of maternal distress, and there was no evidence of a negative bias that may have affected the main results.
